# One landscape does not fit all: Diverse arthropod responses to land use

**DOI:** 10.1002/eap.70132

**Published:** 2025-11-12

**Authors:** Mia K. Lippey, Jay A. Rosenheim, Daniel Paredes, Daniel S. Karp, Sara E. Emery, Rebecca Chaplin‐Kramer, Richard Sharp, Emily K. Meineke

**Affiliations:** ^1^ Department of Entomology and Nematology University of California, Davis Davis California USA; ^2^ Centre for Functional Ecology, Associated Laboratory TERRA, Department of Life Sciences University of Coimbra Coimbra Portugal; ^3^ Department of Wildlife, Fish, and Conservation Biology University of California, Davis Davis California USA; ^4^ Department of Entomology Cornell AgriTech Geneva New York USA; ^5^ World Wildlife Fund Global Science Stanford California USA

**Keywords:** agroecology, conservation biological control, crop pests, crop production, ecoinformatics, landscapes, natural enemies, pesticides

## Abstract

Early studies and theory suggested that complex landscapes harboring remnants of natural land should support natural enemy populations and reduce pest buildup in adjacent crops, whereas landscapes interspersed with urban land often provide alternate host plants of crop pests, facilitating pest spillover and amplifying pest pressure. However, recent meta‐analyses have demonstrated that both pest and beneficial agricultural arthropods respond inconsistently to surrounding landscapes. These meta‐analyses relied on studies of one to two pests per crop across many different crop and landscape contexts, which limits inferences about how growers might design landscapes for effective control of a full suite of pests attacking a given crop. Here, we harnessed an ecoinformatics dataset from California *Citrus* to examine the effects of surrounding natural and urban land on the densities of a complete suite of seven major pest species (6489 observations) and one beneficial predator (346 observations). We also explored landscape effects on pesticide use and fruit production. Despite restricting this analysis to data collected in the same region and cropping system, we found that arthropods still exhibited mixed responses to surrounding landscapes. Among the eight *Citrus*‐associated arthropods surveyed, greater amounts of nearby natural land resulted in two beneficial outcomes for farmers (lower pest densities or fewer pesticide applications targeting that pest), three adverse outcomes, and three neutral outcomes. Similarly, greater amounts of urban land resulted in two beneficial outcomes, four adverse outcomes, and two neutral outcomes for farmers. Our economic analysis demonstrated that *Citrus* groves with more nearby natural land resulted in increased total pesticide use and reduced total fruit yield. More urban land resulted in reduced total pesticide use and no effect on total fruit yield. Neither land use type significantly impacted fruit quality. Taken altogether, our results do not demonstrate clear support for the retention of natural habitat or minimization of urban land near cropland solely for the purpose of enhancing conservation biocontrol. Nonetheless, the value of natural land extends far beyond its utility for conservation biocontrol, and agricultural landscapes must still be managed to strike a balance between crop production and the preservation of biodiversity and ecosystem function.

## INTRODUCTION

Crops are often planted near natural habitats, and urban centers are extending ever closer to landscapes that were once predominantly used for agriculture. Several studies have examined the effects of land use on the animals that inhabit changing landscape mosaics, demonstrating that agricultural intensification and expansion at the expense of surrounding natural habitat reduce the diversity of birds, insect pollinators, and other species that provide ecosystem services (Cusser et al., 2016; Kajzer‐Bonk & Nowicki, 2022; Potts et al., 2010; Shi et al., 2021). Similarly, a large body of literature has demonstrated negative effects of urbanization on non‐pest animal density and diversity (Chamberlain et al., 2009; Fenoglio et al., 2021; Habel et al., 2024; Kajzer‐Bonk & Nowicki, 2022; Liang et al., 2023; Newbold et al., 2015). Thus, conservation ecologists often discourage the expansion and intensification of both agricultural and urban development to conserve biodiversity and ecosystem services (Eigenbrod et al., 2011; Gonthier et al., 2014).

The effects of adjacent landscape composition on arthropod pest pressure in crops, however, remain unclear. Two long‐held hypotheses arising from agroecological theory are often referenced when considering how agricultural landscapes could be managed to reduce pest pressure through conservation biocontrol via direct and indirect mechanisms. The first hypothesis proposes that unmanaged (hereafter, “natural”) land located in and around crop fields may reduce pest pressure directly by impeding pest dispersal, and performance (“resource concentration hypothesis”; Root, 1973; Wetzel et al., 2016), and indirectly by supporting natural enemy populations that provide top‐down control of pests (Bianchi et al., 2006; Rusch et al., 2016; Veres et al., 2013). Support for the importance of retaining natural habitats adjacent to crop fields emerged from early observations of pest outbreaks in monocultures and simplified landscapes (Landis et al., 2000; Pimentel, 1961; Root, 1973). Since then, several case studies have endorsed the incorporation of natural habitat in and around agriculture (Cottrell & Yeargan, 1999; Milligan et al., 2016; Paredes et al., 2021).

A second non‐mutually exclusive hypothesis proposes that urban land located near crop fields may support pest populations directly through the buildup of insect pests on ornamental plants followed by pest dispersal into cropland (“pest spillover”; Cadotte et al., 2017; Gaertner et al., 2017) and indirectly through reduced density of biological control agents (Korányi et al., 2022). Again, this idea has been supported by case studies, including analyses of the Mediterranean fruit fly (*Ceratitis capitata*) and the Asian citrus psyllid (*Diaphorina citri*), which are more likely to establish and thrive in agricultural fields near urban areas (Bayles et al., 2022; Krasnov et al., 2019).

In direct opposition to the theory that predicts beneficial effects of natural land and harmful effects of urban land on nearby crops, recent meta‐analyses demonstrate that natural and urban landscapes display mixed effects on pest pressure in adjacent agricultural areas. A comprehensive, global meta‐analysis of 132 studies including over 30 species of agricultural arthropods revealed that nearby natural habitat was associated with diverse effects on pest densities, predator densities, crop damage, and crop yield, overall showing no consistent trends (Karp et al., 2018). Similarly, a meta‐analysis demonstrated that increasing urbanization had no significant effect on the overall abundances of pest and beneficial arthropod species (Korányi et al., 2022).

Virtually all studies addressing the influences of natural habitat or urban land use on agricultural pest dynamics have focused on one, or sometimes two, pests attacking a given crop. Thus, it remains largely unknown if the variable effects seen across different studies stem from features of the landscape, the crop, or the pest and natural enemy species involved. Importantly, landscape‐level effects of surrounding land use apply to the entire pest and natural enemy community (Chaplin‐Kramer et al., 2011; Veres et al., 2013). If we control for region and crop type, the key question that remains is: can we generalize the effect of habitat across a diverse pest and natural enemy community? If the effects of surrounding land use on multiple pests or natural enemies within the same cropping system are consistent, then concrete recommendations could be developed to support—or, at least, not hinder—conservation biological control. For instance, managers might be encouraged to retain natural habitats and avoid placing crops near urban areas. If, however, pest and natural enemy responses to surrounding land use are mixed across species within a given crop, then managers may face important trade‐offs that make landscape‐level conservation biocontrol programs complex and potentially impractical. To address this research gap, studies are needed to assess whether diverse pests attacking a single crop in a single region show consistent responses to adjacent land use.

Further, the population dynamics of arthropods studied in crop fields may not translate into predictable outcomes for growers across all measures of crop production (Gagic et al., 2019). For example, pest pressure is likely to influence measures of crop production such as total pesticide use and fruit quality, because pesticide use is a direct reflection of pest densities, and much of the downgrading of damaged fruit is caused by pest feeding. In contrast, pest pressure may not always influence total crop yield, because growers generally prevent pest populations from building up beyond the point of yield loss. Instead, crop yield has been tightly linked to soil quality (D'Hose et al., 2014; Suresh & Nagesh, 2015), a feature of the agricultural matrix that varies non‐randomly across the landscape (Poveda et al., 2018; Verburg et al., 2004). Thus, the successful implementation of research‐driven recommendations also hinges upon examination of landscape effects on downstream economic measures for growers (Chaplin‐Kramer et al., 2019; Guerry et al., 2015).

Here, we harness a large database of arthropod densities collected by pest control advisors working in commercial *Citrus* farming in the San Joaquin Valley of California to explore how the proportion of surrounding natural and urban land affects different species in a diverse arthropod pest community. We also assessed the consequences of land use on total pesticide use, fruit quality, and total fruit yield, all of which are metrics of direct economic importance for growers (Chaplin‐Kramer et al., 2019). From this database, we analyzed data from 505 commercial *Citrus* fields between the years 2004 and 2019. Data included arthropod densities (sample sizes varied from 346 to 1424 field‐years across species), pesticide use for 1937 field‐years, fruit quality observations for 1483 field‐years, and total fruit yield for 1646 field‐years. We tested the predictions that (i) the proportion of natural and urban land would demonstrate contrasting effects on pests, with natural land reducing pest pressure and urban land increasing it (Korányi et al., 2022; Landis et al., 2000), and (ii) a predator species would demonstrate responses that were the inverse of those predicted for pests, with natural land increasing predator density and urban land reducing it (Korányi et al., 2022; Rusch et al., 2016).

## MATERIALS AND METHODS

### Study system

**FIGURE 1:**
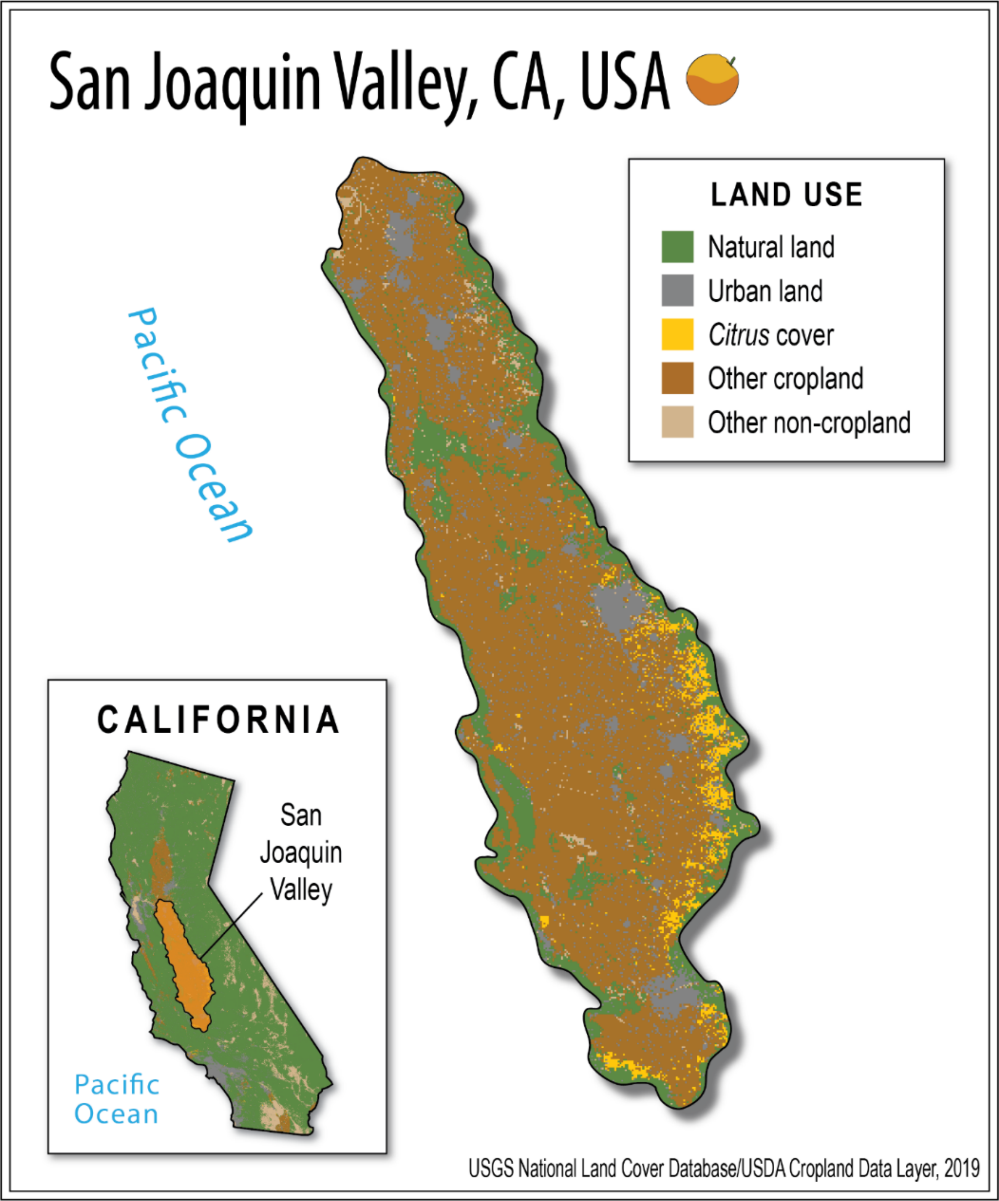
Map of the San Joaquin Valley, California, USA. *Citrus* production (yellow) in the San Joaquin Valley is surrounded by a diversity of land uses, providing an ideal opportunity for studying the roles of natural and urban habitat on pest dynamics in adjacent cropland. Natural land (green) in the San Joaquin Valley occurs along the eastern edge of the valley throughout the foothills of the Sierra Nevada Mountains, characterized predominantly by oak woodland. Urban land (gray) in the region is interspersed throughout cropland (dark brown), comprising various native and non‐native ornamental plants. Other land uses such as water, pasture, and barren land make up the remaining category, other non‐cropland (tan). Land use maps were sourced from the USGS National Land Cover Database (MRLC Consortium, 2023) and the USDA Cropland Data Layer (USDA NASS, 2023). All digital assets were created by Mia K. Lippey using Adobe Illustrator (2024).

We analyzed data from 505 commercial *Citrus* fields in the San Joaquin Valley, California, spanning a latitudinal range of 43.5 km across Tulare, Fresno, and Kern Counties. The area is characterized by a Mediterranean climate with an average temperature of 32°C in summer and 7°C in winter, and an average annual precipitation of 30 cm falling primarily in winter (National Weather Service, US Department of Commerce). *Citrus* production in the San Joaquin Valley is surrounded by a diversity of land uses, providing an ideal opportunity for studying the roles of natural and urban habitat on pest dynamics in adjacent cropland (Figure 1). *Citrus* fields are monocrops, but the San Joaquin Valley produces a diversity of agricultural products, and thus *Citrus* in this region is interdigitated with a diversity of crops (e.g., grapes, plums, almonds, pistachios, olives). Most *Citrus* farms in the region are established along the eastern edge of the valley, near the foothills of the Sierra Nevada Mountains, to provide cold air drainage and reduce the risk of crop freeze damage. As a result, *Citrus* fields vary in their proximity to natural land, characterized predominantly by oak woodland and other plants (see Appendix S1: Table S1). Additionally, the rivers that flow westward out of the Sierra Nevada attracted early settlers and growers to the eastern side of the San Joaquin Valley, resulting in the establishment of many communities throughout the area. These urban areas are now dispersed between and around focal *Citrus* fields and include various native and non‐native ornamental plants (see Appendix S1: Table S1).

### Database

We worked with arthropod density and field‐management data stored in a SQL server database that we accessed using a custom‐designed software interface (*Citrusformatics*, Ten2Eleven Business Solutions, LLC, n.d.). Our dataset brings together pest control advisor reports and grower‐provided data on commercial *Citrus* production from 11 growers. Among the contributing growers, one large commercial grower supplied 67% of our data (268 *Citrus* fields), with the other 10 independent growers together contributing the remaining 33% (ca. 24 *Citrus* fields per grower on average). Scouting records include estimates of pest and predator densities, with each species sampled using different methods to estimate either abundance or percent crop infestation (Table 1, Appendix S2). Additionally, since different growers sometimes used different methods to sample the same species, for each species we used only subsets of data generated with the same sampling methods. Analyses thus included density estimates for seven pest species and one predatory mite genus *Euseius*, consisting primarily of the native species *Euseius tularensis*. Field observations were available for 6489 field‐year observations spanning the years 2004–2019.

**TABLE 1:** The eight focal arthropod species selected for our analysis using the *Citrusformatics* database, including seven pest species and one beneficial mite.

Species	Description	Sampling method	No. observations (field‐years)
Fork‐tailed bush katydid (*Scudderia furcata*)	Pest; omnivore	Presence/absence sampling on *Citrus* trees	714
Citricola scale (*Coccus pseudomagnoliarum*)	Pest; generalist herbivore	Presence/absence sampling on *Citrus* trees	766
California red scale (*Aonidiella aurantii*)	Pest; generalist herbivore	Count of infested fruit at harvest	765
Citrus thrips (*Scirtothrips citri*)	Pest; generalist herbivore	Presence/absence sampling on *Citrus* trees	1424
Citrus red mite (*Panonychus citri*)	Pest; generalist herbivore	Presence/absence sampling on *Citrus* trees	1130
Cottony cushion scale (*Icerya purchasi*)	Pest; generalist herbivore	Presence/absence sampling on *Citrus* trees	592
Citrus peelminer (*Marmara gulosa*)	Pest; generalist herbivore	Count of damaged fruit at harvest	752
*Euseius* mite (*Euseius* spp.)	Natural enemy; generalist predator	Count of individuals on *Citrus* trees	346

*Note*: All of the focal arthropod species are generalist herbivores with the exception of one omnivorous pest species (fork‐tailed bush katydid) and one predatory mite (*Euseius* spp.) (see Appendix S2). Though sampling methods vary by arthropod species, all methods estimate a measure of population density.

Grower records in the database include targeted pesticide applications (for each focal pest species), total pesticide use (including non‐focal pest species), fruit quality, and total fruit yield (Table 2). We took various approaches to prepare these data for analysis. Pesticide applications were occasionally (but rarely) applied to less than the full field, resulting in some non‐integer values in the raw data; these values were rounded to the nearest integer. To justify rounding values and fitting Poisson distributions, we also modeled pesticide data by excluding observations with non‐integer values, and results did not vary significantly between the two approaches. Fruit quality data were converted to a single, continuous quality metric ranging from 0 to 2. To do this, we first grouped *Citrus* fruit classes into three quality categories using reports from packinghouses (i.e., low quality = rot/juice classes, medium quality = choice class, high quality = premium/fancy/export classes, but note that some fruit classes vary by *Citrus* type; Table 2). We then calculated a weighted average across the harvest by multiplying each category by an integer value between 0 and 2, where low quality was multiplied by zero, medium quality by one, and high quality by two, and final values were summed per *Citrus* field.

**TABLE 2:** The pesticide use and crop production metrics selected for analysis using the *Citrusformatics* database.

Response variable	Description	Original metric/units	Final metric/units	No. observations
Targeted pesticide applications	No. targeted pesticide sprays reported per *Citrus* field for each focal pest species	Quantitative, reported as non‐integer counts	Integer count	7630
Total pesticide use	Total no. pesticide sprays reported per *Citrus* field, including sprays for non‐focal arthropod pests (i.e. ants, snails, earwigs, cutworms, and the orange dog caterpillar)	Quantitative, reported as non‐integer counts	Integer count	1937
Fruit quality	Quality of *Citrus* fruits assigned at harvest per *Citrus* field	Qualitative, reported as categorical classes	Continuous ranking (0–2)	1483
Total fruit yield	Total fresh mass of *Citrus* fruit recorded at harvest per *Citrus* field	Quantitative, reported as pounds	Kilograms per hectare	1646

*Note*: To prepare these data, we performed a range of data transformations.

### Land use data

We gathered land use data around each focal *Citrus* field from two remotely sensed sources. The primary source we used to determine the amount of natural and urban land was the National Land Cover Database (NLCD) developed by the MultiResolution Land Characteristics Consortium (MRLC Consortium, 2023). Because the NLCD does not distinguish between specific crop types, we also used the Cropland Data Layer (CDL) developed by the USDA National Agricultural Statistics Service (USDA NASS, 2023) to determine the amount of *Citrus* cover in the surrounding landscape.

Our original intention was to use the CDL to test the resource concentration hypothesis focusing on surrounding *Citrus* fields, alongside our hypotheses regarding natural and urban land. We aimed to determine if greater *Citrus* surrounding our focal *Citrus* fields would increase pest buildup. However, ground‐truthing revealed that the CDL misidentified neighboring crops in 69% of the 58 crop fields we assessed. We therefore relied on NLCD for the majority of land use data collection, as it is considered highly accurate and is commonly applied in landscape‐scale studies (Emery et al., 2024; Gutiérrez Illán et al., 2020; Janvier et al., 2022; Larsen & McComb, 2021). Due to the CDL's inaccuracy at identifying particular crops, we used it solely to estimate surrounding *Citrus* cover as a covariate.

Land use studies inherently involve trade‐offs between different landscape elements: when one land use type decreases, another increases, because land use area must sum to 1.0. In this particular system, if natural land around a focal *Citrus* grove decreases, it is likely cropland that increases (and likewise for urban land). By controlling for *Citrus* area, we can attribute observed effects to changes in natural and urban land coverage rather than to variation in *Citrus* area; *Citrus* could be especially important, as it is a host plant resource.

Within the years relevant to the *Citrusformatics* database, NLCD land use data were available for the years 2004, 2006, 2008, 2011, 2013, 2016, and 2019. We linearly interpolated land use data across missing years to create 16 continuous data layers (2004–2019). We first categorized NLCD land uses into four groups: natural land, urban land, cropland, and other non‐cropland. Natural land included shrub/scrub, herbaceous plants, woody wetlands, and emergent herbaceous wetlands. Urban land comprised developed space (low‐, medium‐, and high‐intensity developed areas). Cropland consisted of the single NLCD category “cultivated crops.” Other non‐cropland included hay/pasture, barren land, and open water. We then used the CDL to calculate the proportion of *Citrus* crop cover surrounding our focal *Citrus* fields by combining two CDL categories: oranges and *Citrus*. To determine our final land use category of “other cropland,” we subtracted the amount of *Citrus* cover from the NLCD measure of “cultivated cropland.” For our final dataset, we ultimately delineated five land use categories: (1) natural, (2) urban, (3) *Citrus*, (4) other cropland, and (5) other non‐cropland (Figure 1, Appendix S3).

Using ArcGIS, we quantified the proportions of natural, urban, *Citrus*, other cropland, and other non‐cropland within multiple circular buffers surrounding focal *Citrus* fields between the years 2004 and 2019. We subtracted the focal *Citrus* field from each circular buffer centered on each focal *Citrus* field. The proportions of land use types were calculated using the number of pixels of each land type divided by the total buffer area. Because the biologically relevant spatial scale for each of the studied arthropods was unknown, we examined the effects of surrounding land use at four spatial scales (i.e., buffer radii of 500 m, 1 km, 2 km, and 4 km).

### Modeling pest and predator densities

All analyses were conducted using the statistical software R version 4.3.2 (R Core Team, 2023). To control for spatial autocorrelation, the study region was divided into 16 equal‐sized subregions using latitude and longitude. The subregion number was included in the model as a categorical variable and assigned as a random intercept. We ran a Moran's I analysis to ensure that spatial autocorrelation was sufficiently mitigated by the subregion model term (Appendix S4). Across all spatial scales and analyses performed, the variance inflation factor for the three land use types (natural land, urban land, and *Citrus*) remained between 1.00 and 1.21, so all three predictors were included in the same statistical models. We dropped two land use categories, other cropland and other non‐cropland, from the model to avoid nonindependence between land use categories (the five land use proportions must sum to 1.0) (see Appendix S3: Table S1).

Pest and predator densities in response to land use were modeled individually by arthropod species. Field size was included as a covariate. The unique field identification number (to control for multiple observations in the same field), the subregion (to address issues of spatial autocorrelation), and the year were included as random effects:

$$\text{Arthropod population density}\sim \text{urban land}+\text{natural land}+\text{Citrus cover}+\text{field size}+\left(1|\text{unique field}\;\mathrm{ID}\right)+\left(1|\text{subregion}\right)+\left(1|\text{year}\right).$$

All species were modeled using generalized linear mixed models (*glmmTMB* R package, Brooks et al., 2017). We used the default model‐based SEs provided by the *glmmTMB* package, which are derived from the inverse of the observed Fisher Information Matrix. Due in part to the different sampling methods used across arthropod species (Table 1, Appendix S2), the response variables included in the analyses varied in distributional characteristics. We therefore tailored our models to each species by applying appropriate error families: five species (fork‐tailed bush katydid, citricola scale, citrus red mite, cottony cushion scale, and citrus thrips) were fit with a zero‐inflated beta distribution (beta_family with zi ~1, a *glmmTMB* option), two species (California red scale and citrus peelminer) were better fit by a zero‐inflated Tweedie distribution (Tweedie with zi ~1, a *glmmTMB* option), and one species (*Euseius* mite) was fit with a simple Tweedie distribution. Distributions for each model were checked using the *DHARMa* package, and all model assumptions were closely met (*DHARMa* R package, Hartig, 2022). We report modeling results for the spatial scale that minimized the Akaike information criterion (AIC) value for each species (see Appendix S5: Table S1).

To facilitate visual comparisons across species, pest and predator density plots are shown using ggpredict() in R to display the linear beta coefficient of regression for natural and urban land from each species model, which plots the partial effect of land use proportions while holding all other predictors at their mean values and random effects at zero. While *glmmTMB* models accommodate nonlinear relationships, the coefficients reported for each predictor variable represent the linear effect of these predictors on the link‐transformed response. The CI ribbons remain nonlinear to show the uncertainty estimates of the true relationship between land use and species density. For the zero‐inflated beta models (fork‐tailed bush katydid, citricola scale, citrus red mite, cottony cushion scale, and citrus thrips), we are reporting the coefficients from the beta component (not the zero‐inflation component), which estimate the effect of predictors on the mean of the distribution for nonzero values. These coefficients represent the change in the logit‐transformed mean of the beta distribution per unit change in the predictor. For the zero‐inflated Tweedie (California red scale and citrus peelminer) and simple Tweedie models (the predatory *Euseius* mite), the coefficients represent the effect of predictors on the log‐transformed mean of the Tweedie distribution. Reported percent changes are calculated as the relative difference in predicted values across the full range of land use proportions (from minimum to maximum observed values) using the predict() function in R, which preserves the random effect structure of the models, and are expressed as percentage changes relative to the mean prediction.

### Modeling targeted pesticide applications

Possible effects of landscape composition on pest population dynamics could be expressed either through changes in observed pest densities or changes in the frequency of pesticide applications, because pesticide applications are generally responsive to observed pest densities. We therefore modeled the effect of land use on the number of pesticide applications targeting each of our focal pests. The causal relationship between pest density and pesticide use is potentially bidirectional: pest density affects the decision to initiate pesticide applications and pesticide applications can suppress target pest populations. As a result, pesticide applications could not be included as a predictor in the model for pest density. Instead, we ran separate models to examine the number of pesticide applications and interpreted the results alongside the results of the pest density models (Rosenheim et al., 2022).

Pesticide applications were targeted to particular pests; therefore, associations between pesticides and surrounding land uses were also modeled individually by arthropod species. Again, field size was included as a covariate, and unique field identification number, subregion, and crop year were included as random effects. Three species were excluded from the pesticide analysis, because they were either never the target of pesticide applications (“citrus peelminer”), only very rarely the target of applications (“cottony cushion scale”), or represented a beneficial predator (“*Euseius* spp. mite”). Targeted pesticide application data were fit using a Poisson distribution and modeled using *glmmTMB* R package (Brooks et al., 2017). We report modeling results at the same scale as that which minimized the AIC value for associated pest densities (see Appendix S5: Table S2).

Targeted pesticide application plots are also shown using ggpredict() in R to display the linear beta coefficient of regression for natural and urban land from each model, and CI ribbons remain nonlinear. For the Poisson models (all targeted pesticide models), the Poisson family in *glmmTMB* automatically implements a log link function. The slopes in these models represent the effect on the log‐transformed mean count of pesticide applications per unit change in the predictor. Reported percent changes are calculated the same way as the density model results.

### Modeling total pesticide use, fruit quality, and total fruit yield

The economic measures of crop production were modeled the same way with field size included as a covariate, and unique field identification number, subregion, and crop year included as random effects. For total fruit yield, tree age was also included as a polynomial covariate as *Citrus* trees do not reach full production capacity for several years after planting. Again, these data varied in distributional characteristics and thus required a variety of statistical approaches, but all response variables were modeled using a *glmmTMB* R package (Brooks et al., 2017). Total pesticide use data were fit using a Poisson distribution. Fruit quality data, once converted to a continuous quality metric from zero to two, were rescaled and transformed to fit a beta distribution (beta_family [link = logit], a *glmmTMB* option). Total yield data were fit with a simple Tweedie distribution. We report modeling results for the spatial scale that minimized AIC values for each response variable (see Appendix S5: Table S3).

Again, figures are shown using ggpredict() in R to display the linear beta coefficient of regression for natural and urban land from each model, and CI ribbons remain nonlinear. For the Poisson models (total pesticide use), the beta models (fruit quality), and the simple Tweedie models (total fruit yield), the coefficients represent the effects of predictors as described above for arthropod density and targeted pesticide models. Reported percent changes are calculated the same way as the density and targeted pesticide application results.

## RESULTS

### Pest and predator densities

**FIGURE 2:**
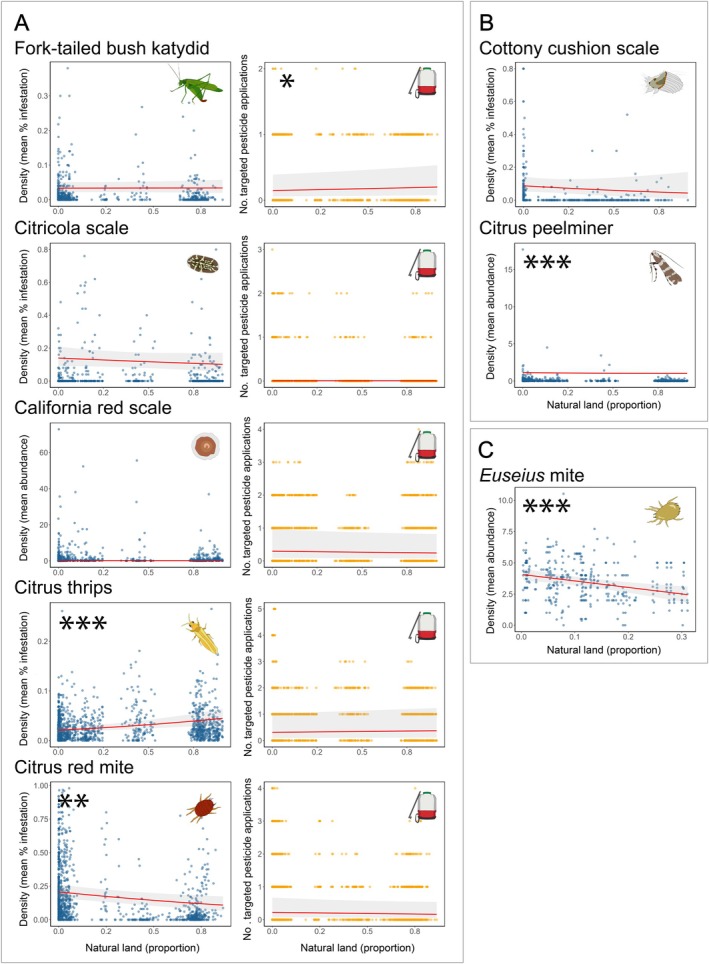
Effects of surrounding natural land on eight focal arthropod species in *Citrus* groves in the San Joaquin Valley. Natural land (*x*‐axis) is quantified as proportional land cover within a circular buffer surrounding each focal *Citrus* grove. Variation in natural land distribution between species reflects sampling inconsistencies across farms, with uniformly sampled species showing complete distributions while others display gaps where only consistently sampled subsets could be used. Each density point (blue) represents the mean observation per *Citrus* field for a single year. Each targeted pesticide application point (orange) represents the number of pesticide sprays per *Citrus* field for a single year. Asterisks indicate the level of statistical significance (*p < 0.05; **p < 0.01; ***p < 0.001), and 95% CIs (shaded area) are shown. All results are reported at a spatial scale of 4 km with the exception of the fork‐tailed bush katydid (2 km), citrus red mite (2 km), and cottony cushion scale (500 m). Panel (A) shows paired response variables of density observations and targeted pesticide applications for five species: fork‐tailed bush katydid (effect of natural land on density [generalized linear mixed model (GLMM) zero‐inflated beta family; *n* = 714, β = 0.006, *p* = 0.936] and on targeted pesticide applications [GLMM Poisson family; *n* = 1526, β = 0.114, *p* = 0.019]), citricola scale (effect of natural land on density [GLMM zero‐inflated beta family; *n* = 766, β = −0.146, *p* = 0.292] and on targeted pesticide applications [GLMM Poisson family; *n* = 1526, β = −0.030, *p* = 0.780]), California red scale (effect of natural land on density [GLMM zero‐inflated Tweedie family; *n* = 765, β = −0.229, *p* = 0.433] and on targeted pesticide applications [GLMM Poisson family; *n* = 1526, β = −0.078, *p* = 0.294]), citrus thrips (effect of natural land on density [GLMM zero‐inflated beta family; *n* = 1424, β = 0.302, *p* = 0.000] and on targeted pesticide applications [GLMM Poisson family; *n* = 1526, β = 0.068, *p* = 0.051]), and citrus red mite (effect of natural land on density [GLMM zero‐inflated beta family; *n* = 1130, β = −0.271, *p* = 0.003] and on targeted pesticide applications [GLMM Poisson family; *n* = 1526, β = −0.110, *p* = 0.267]). Panel (B) shows only density observations for two species for which pesticides are rarely applied: cottony cushion scale (effect of natural land on density [GLMM zero‐inflated beta family; *n* = 592, β = −0.198, *p* = 0.335]), and citrus peelminer (effect of natural land on density [GLMM zero‐inflated Tweedie family; *n* = 752, β = −0.902, *p* = 0.000]). Panel (C) shows density observations for the predatory *Euseius* mite for which pesticides are not applied and interpretations of landscape effects are the inverse of pests (effect of natural land on density [GLMM Tweedie family; *n* = 436, β = −0.140, *p* = 0.000]). All digital assets were created by Mia K. Lippey using Adobe Illustrator (2024).

We found mixed effects of urban and natural land on densities of the seven *Citrus*‐associated pests. Natural land was significantly associated with higher densities of one pest (citrus thrips) and lower densities of two pests (citrus red mite and citrus peelminer) and had no significant association with density for four pests (fork‐tailed bush katydid, citricola scale, California red scale, and cottony cushion scale) (Figure 2). Models predicted that as the proportion of surrounding natural land increases from 0.0 to 1.0, citrus thrips densities would increase by 114% from 2.1% to 4.5% infestation. In contrast, citrus red mite would decrease by 47% from 20.7% to 10.9% infestation, and citrus peelminer densities would decrease by 9% from 1.1 to 1.0 damaged fruits per bin at harvest.

**FIGURE 3:**
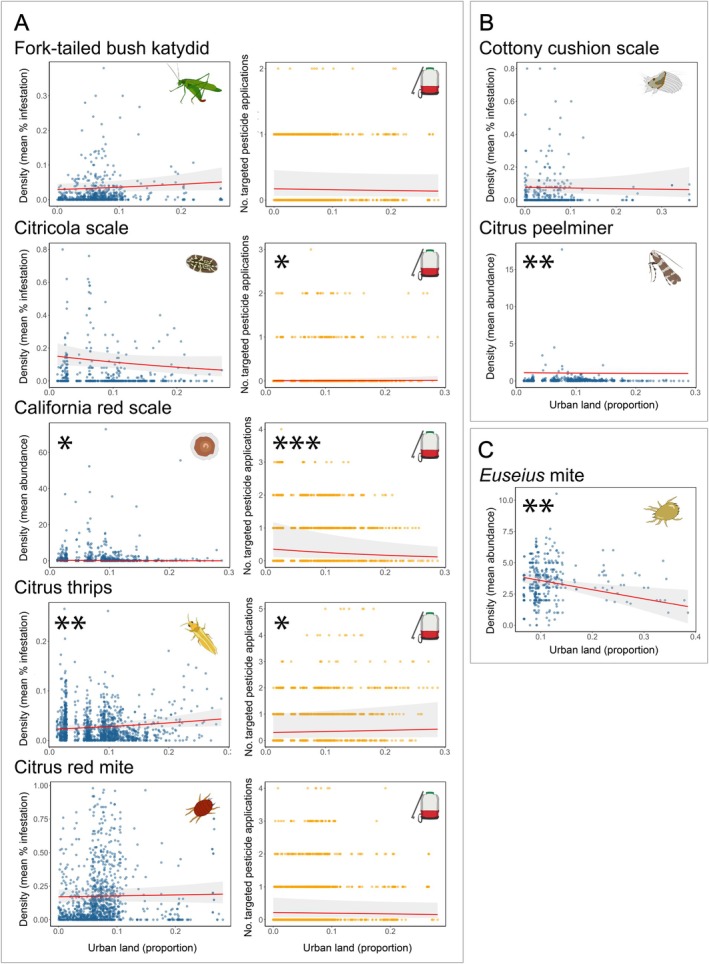
Effects of surrounding urban land on eight focal arthropod species in *Citrus* groves in the San Joaquin Valley. Urban land (*x*‐axis) is quantified as proportional land cover within a circular buffer surrounding each focal *Citrus* grove. Variation in urban land distribution between species reflects sampling inconsistencies across farms, with uniformly sampled species showing complete distributions while others display gaps where only consistently sampled subsets could be used. Each density point (blue) represents the mean observation per *Citrus* field for a single year. Each targeted pesticide application point (orange) represents the number of pesticide sprays per *Citrus* field for a single year. Asterisks indicate the level of statistical significance (*p < 0.05; **p < 0.01; ***p < 0.001), and 95% CIs (shaded area) are shown. All results are reported at a spatial scale of 4 km with the exception of the fork‐tailed bush katydid (2 km), citrus red mite (2 km), and cottony cushion scale (500 m). Panel (A) shows paired response variables of density observations and targeted pesticide applications for five species: fork‐tailed bush katydid (effect of urban land on density [generalized linear mixed model (GLMM) zero‐inflated beta family; *n* = 714, β = 0.097, *p* = 0.074] and on targeted pesticide applications [GLMM Poisson family; *n* = 1526, β = −0.039, *p* = 0.423]), citricola scale (effect of urban land on density [GLMM zero‐inflated beta family; *n* = 766, β = −0.185, *p* = 0.106] and on targeted pesticide applications [GLMM Poisson family; *n* = 1526, β = 0.200, *p* = 0.010]), California red scale (effect of urban land on density [GLMM zero‐inflated Tweedie family; *n* = 765, β = −0.431, *p* = 0.023] and on targeted pesticide applications [GLMM Poisson family; *n* = 1526, β = −0.202, *p* = 0.001]), citrus thrips (effect of urban land on density [GLMM zero‐inflated beta family; *n* = 1424, β = 0.121, *p* = 0.010] and on targeted pesticide applications [GLMM Poisson family; *n* = 1526, β = 0.064, *p* = 0.041]), and citrus red mite (effect of urban land on density [GLMM zero‐inflated beta family; *n* = 1130, β = 0.023, *p* = 0.639] and on targeted pesticide applications [GLMM Poisson family; *n* = 1526, β = −0.053, *p* = 0.260]). Panel (B) shows only density observations for two species for which pesticides are rarely applied: cottony cushion scale (effect of urban land on density [GLMM zero‐inflated beta family; *n* = 592, β = −0.031, *p* = 0.750]), and citrus peelminer (effect of urban land on density [GLMM zero‐inflated Tweedie family; *n* = 752, β = −0.416, *p* = 0.002]). Panel (C) shows density observations for the predatory *Euseius* mite for which pesticides are not applied and interpretations of landscape effects are the inverse of pests (effect of urban land on density [GLMM Tweedie family; *n* = 346, β = −0.127, *p* = 0.003]). All digital assets were created by Mia K. Lippey using Adobe Illustrator (2024).

Urban land was significantly associated with higher densities of one pest (citrus thrips) and lower densities of two pests (California red scale and citrus peelminer) and had no significant association with density for four pests (fork‐tailed bush katydid, citricola scale, citrus red mite, and cottony cushion scale) (Figure 3). Models predicted that as the proportion of surrounding urban land increases from 0.0 to 0.3, citrus thrips densities would increase by 87% from 2.3% to 4.3% infestation. In contrast, California red scale would decrease by 50% from 0.2 to 0.1 damaged fruits per bin at harvest, and citrus peelminer densities would decrease by 9% from 1.1 to 1.0 damaged fruits per bin at harvest, respectively.

For the one beneficial arthropod, the predatory *Euseius* mite, we found that both natural and urban land were significantly associated with decreases in density (Figures 2 and 3). Models predicted that as the proportion of surrounding natural and urban land increases from 0.0 to 0.3, *Euseius* mite densities would decrease by 39% from 4.1 to 2.5 individuals and 61% from 3.8 to 1.5 individuals, respectively.

### Targeted pesticide applications

We also found mixed effects of urban and natural land on the number of targeted pesticide applications. Natural land was significantly associated with higher numbers of pesticide applications targeting one pest (fork‐tailed bush katydid) and lower numbers of pesticide applications for no pests and had no significant association with numbers of targeted pesticide applications for four pests (citricola scale, citrus thrips, California red scale, and citrus red mite) (Figure 2). Models predicted that as the proportion of surrounding natural land increases from 0.0 to 1.0, targeted pesticide applications for fork‐tailed bush katydid would increase by 67% from 0.3 to 0.5 sprays.

Urban land was significantly associated with higher numbers of pesticide applications targeting two pests (citricola scale, and citrus thrips) and lower numbers of targeted pesticide applications of one pest (California red scale) and had no significant association with numbers of targeted pesticide applications for two pests (fork‐tailed bush katydid and citrus red mite) (Figure 3). Models predicted that as the proportion of surrounding urban land increases from 0.0 to 0.3, targeted pesticide applications for citricola scale would increase by 100% from 0.1 to 0.2 sprays. For citrus thrips, they would increase by 33% from 0.9 to 1.2 sprays. In contrast, targeted pesticide applications for California red scale would decrease by 60% from 1.0 to 0.4 sprays.

### Economic measures of *Citrus* production

**FIGURE 4:**
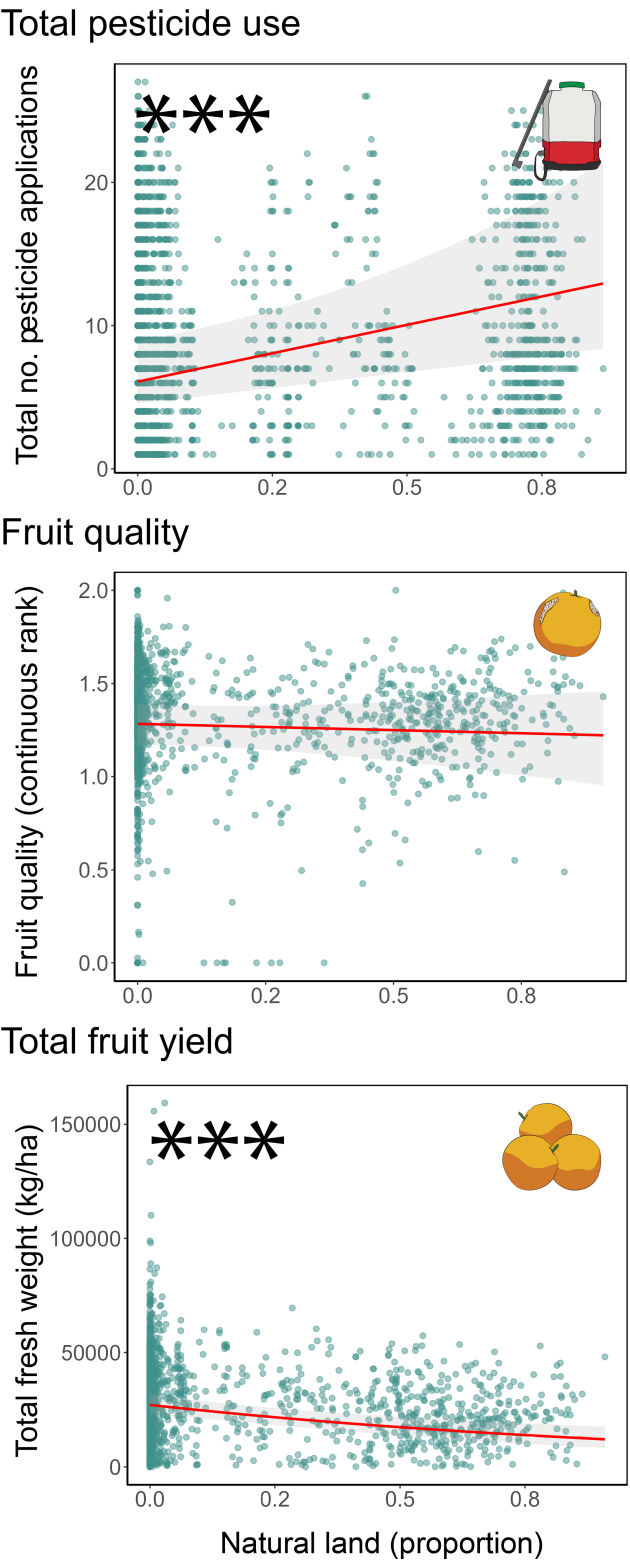
Effects of surrounding natural land on total pesticide use, fruit quality, and total fruit yield in *Citrus* groves across the San Joaquin Valley. Natural land is quantified as proportional land cover within a circular buffer surrounding each focal *Citrus* grove. Each total pesticide use point represents the total number of pesticide applications for all arthropod pest species per *Citrus* field for a single year. Each fruit quality point represents the proportion of low‐, medium‐, and high‐quality fruit (converted to a continuous ranking from zero to two) at harvest per *Citrus* field for a single year. Each total fruit yield point represents the total fresh mass of harvested fruit (in kilograms per hectare) per *Citrus* field for a single year. *p < 0.05; 95% CIs (shaded area) are shown. Results for total pesticide use and fruit quality are reported at a spatial scale of 1 km. Total fruit yield is reported at 2 km. Plots show three economic response variables: total pesticide use (effect of natural land [generalized linear mixed model (GLMM) Poisson family; *n* = 1937, β = −0.014, *p* = 0.931]), fruit quality (effect of natural land [GLMM beta family; *n* = 1483, β = −0.144, *p* = 0.635]), and total fruit yield (effect of natural land [GLMM Tweedie family; *n* = 1647, β = −0.357, *p* = 0.000]). All digital assets were created by Mia K. Lippey using Adobe Illustrator (2024).

**FIGURE 5:**
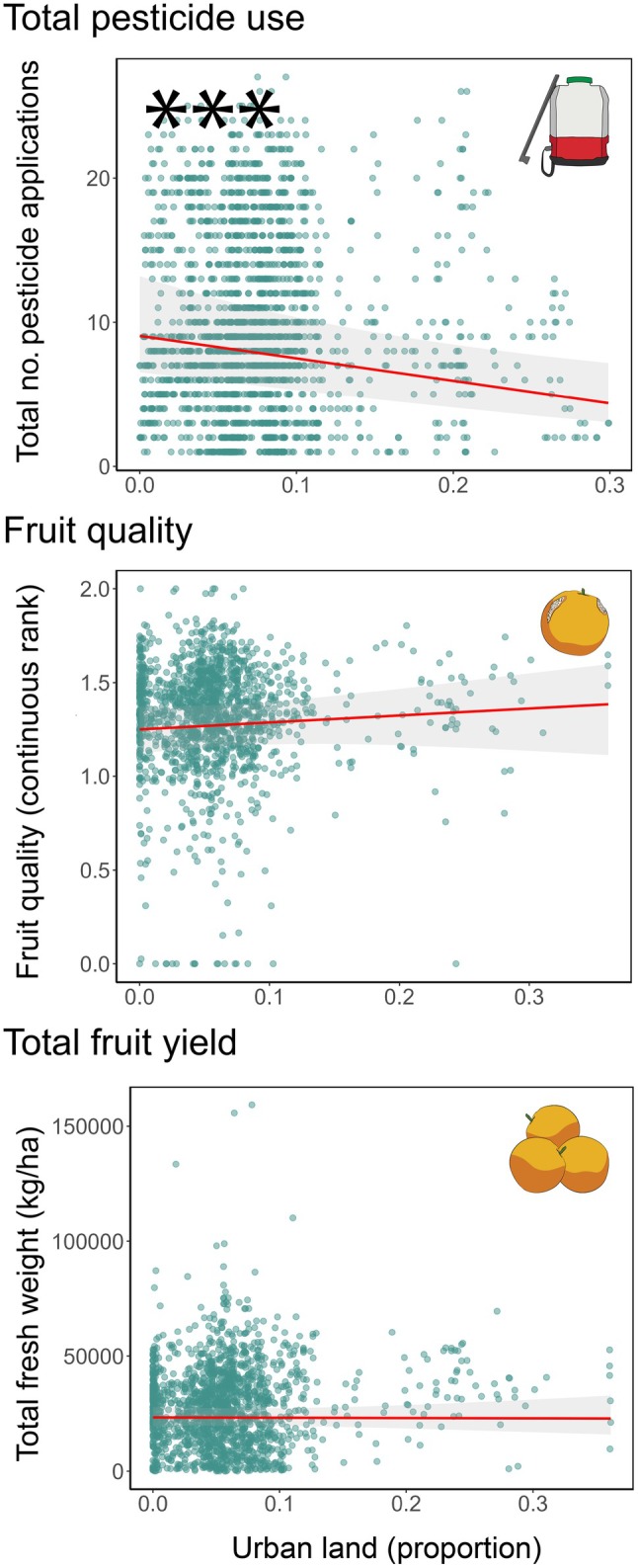
Effects of surrounding urban land on total pesticide use, fruit quality, and total fruit yield in *Citrus* groves across the San Joaquin Valley. Urban land is quantified as proportional land cover within a circular buffer surrounding each focal *Citrus* grove. Each total pesticide use point represents the total number of pesticide applications for all pest species per *Citrus* field for a single year. Each fruit quality point represents the proportion of low‐, medium‐, and high‐quality fruit (converted to a continuous ranking from zero to two) at harvest per *Citrus* field for a single year. Each total fruit yield point represents the total fresh mass of harvested fruit (in kilograms per hectare) per *Citrus* field for a single year. 95% CIs (shaded area) are shown. Results for total pesticide use and fruit quality are reported at a spatial scale of 1 km. Total fruit yield is reported at 2 km. Plots show three economic response variables: total pesticide use (effect of urban land [generalized linear mixed model (GLMM) Poisson family; *n* = 1937, β = 0.655, *p* = 0.095]), fruit quality (effect of urban land [GLMM beta family; *n* = 1483, β = 0.817, *p* = 0.366]), and total fruit yield (effect of urban land [GLMM Tweedie family; *n* = 1647, β = −0.032, *p* = 0.379]). All digital assets were created by Mia K. Lippey using Adobe Illustrator (2024).

Natural land was not associated with harvested fruit quality but was associated with an increase in total pesticide use and a reduction in total fruit yield (Figure 4). The model predicted that as the proportion of surrounding natural land increases from 0 to 1.0, total pesticide use would increase 109% from 5.6 to 11.7 sprays. Total fruit yield would decrease by 55% from 27,089 to 12,055 kg/ha. Urban land was significantly associated with only one measure of *Citrus* production, a reduction in total pesticide use (Figure 5). The model predicted that as the proportion of surrounding urban land increases from 0 to 0.3, total pesticide use would decrease by 49% from 8.1 to 4.1 sprays.

## DISCUSSION

Repurposed pest monitoring data from California *Citrus* in the San Joaquin Valley demonstrate that arthropods respond in diverse ways to surrounding land use. Our results align with meta‐analyses demonstrating no consistent trend in the net effects of either natural land use or urban land use on arthropod densities and targeted pesticide applications (Karp et al., 2018; Korányi et al., 2022). We also found that increasing amounts of natural habitat in the surrounding landscape were associated with increased total pesticide use and reduced fruit yield, while urban land was associated with reduced total pesticide use.

### Net arthropod outcomes

In *Citrus* crops, growers apply pesticides adaptively, in that insecticides are only sprayed when pest densities reach levels perceived to be damaging. We therefore considered changes in the frequency of pesticide applications to be an additional indicator of the effects of landscape composition on pest pressure. Accordingly, our interpretation of net pest pressure experienced by the grower considers the effects of landscape on both arthropod densities and targeted pesticide applications. Net beneficial outcomes for the grower include lower pest densities or fewer targeted pesticide applications. Net adverse outcomes for the grower include higher pest densities or increased targeted pesticide applications. A net neutral landscape effect was inferred when surrounding land use has no significant effect on both pest densities and targeted pesticide applications. Interpretations for the beneficial *Euseius* predatory mite are the inverse of these.

Of the eight focal *Citrus*‐associated arthropods surveyed, the effects of natural and urban land on the net outcomes for growers were similar. Natural land located near *Citrus* fields resulted in net beneficial outcomes for growers for two species (citrus red mite and citrus peelminer), net adverse outcomes for three species (fork‐tailed bush katydid, citrus thrips, and *Euseius* mite), and net neutral outcomes for three species (California red scale, cottony cushion scale, and citricola scale). Urban land located near *Citrus* fields resulted in net beneficial outcomes for growers for two species (California red scale and citrus peelminer), net adverse outcomes for three species (citrus thrips, citricola scale, and *Euseius* mite), and net neutral outcomes for three species (fork‐tailed bush katydid citrus red mite, and cottony cushion scale).

Importantly, not all of our focal pests are equally damaging. For a landscape design to effectively benefit farmers, it may not be necessary to accrue benefits across all species; suppressing the most harmful pests may be sufficient. Among our focal species, California red scale, citrus thrips, and fork‐tailed bush katydid cause the most severe economic damage (UC IPM). However, when examining just these three species, results remain mixed for both land use types. Natural land showed adverse effects for two species and neutral effects for one species, while urban land showed beneficial effects for one species, adverse effects for one species, and neutral effects for one species. Thus, even when restricting interpretations to the three most damaging pests, landscape effects remain complex.

Our results for growers highlight the mixed effects of surrounding land use on both arthropod densities and targeted pesticide use, and thus a mixed set of recommendations related to each pest or beneficial species within a single cropping system. This diversity in arthropod density and pesticide responses means that growers cannot readily manage the surrounding landscape to uniformly maximize pest control. These results in *Citrus* support the conclusions of previous meta‐analyses (Karp et al., 2018; Korányi et al., 2022) that arthropod populations have species‐specific responses to land use. We conclude that attempting to design landscapes to optimize control of the entire pest complex in California *Citrus* is unlikely to be successful.

### Effects of surrounding landscapes on pests and predator species

We predicted that (i) the proportion of natural and urban land would demonstrate contrasting effects on pests, with natural land reducing pest pressure and urban land increasing it (Korányi et al., 2022; Landis et al., 2000). Our findings did not support these predictions, as net pest outcomes exhibited a wide range of responses to both land use types. Several life history traits may explain the diversity of arthropod responses to surrounding natural and urban land, particularly diet breadth, overwintering biology, and dispersal ability.

Diverse responses to surrounding land covers may be due in part to the generalist diet of all the focal pests in the *Citrus* community. Unlike pests of annual crops (Tscharntke et al., 2016), pests of perennial crops do not depend on surrounding landscapes for survival and reproduction, as evergreen crops like *Citrus* remain viable host plants year‐round. However, the ability to consume alternate host plants may still influence how each arthropod interacts with the surrounding landscape. These *Citrus* pests have been documented on numerous alternative host plants, ranging from 20 to 111 different plants across pest species (see Appendix S1: Table S2), many found throughout both natural and urban areas in the San Joaquin Valley (see Appendix S1: Table S1). Alternate host plants vary in nutritional quality and effects on population growth (Chen et al., 2023; Kumar et al., 2021; Nguyen‐Dang et al., 2016), and species show different preferences for oviposition on these plants, a key trait for population buildup (Kühnle & Müller, 2011; Silva et al., 2021; Suwarno et al., 2010). Thus, the mixed responses of pests to surrounding natural and urban land are influenced by species‐specific host preferences, the configuration of alternate hosts, and each plant's quality as a resource for each pest.

These diverse responses may also be influenced by species‐specific aspects of overwintering biology. While perennial crop fields provide year‐round habitat, some species may overwinter elsewhere due to greater shelter or reduced disturbance (Landis et al., 2000). Many studies demonstrate the utility of natural habitat remnants and urban greenspaces as sufficient overwintering sites for arthropods (Clem & Harmon‐Threatt, 2021; Dawadi & Sadof, 2022; Ferlauto & Burghardt, 2025; Sivakoff et al., 2013). Though knowledge about species‐specific overwintering strategies remains limited, the variation in species' preferences for nearby natural or urban land can drive a range of outcomes, with some species effectively utilizing surrounding habitats for overwintering, while others simply take refuge directly within *Citrus* fields (Gui & Boiteau, 2010). The mixed responses of arthropods to surrounding land use are therefore likely influenced by both their dietary flexibility and their overwintering habitat preferences.

Scale insect pests were the taxonomic group least affected by landscape composition. In response to natural land, all three scale insects exhibited net neutral responses (citricola scale, California red scale, cottony cushion scale); in response to urban land, one exhibited a net neutral response (Cottony Cushion Scale, n.d.) while the other two displayed divergent patterns—California red scale showed a net beneficial effect and citricola scale showed a net adverse effect. Unlike pests that actively move through the landscape to exploit alternate habitats, the predominantly neutral responses of scale insects may be explained by their distinctive life cycle. These pests remain sessile for most of their life cycle (Bāders et al., 2018; Karungi et al., 2015; Willard, 1974), severely limiting their ability to colonize new areas during this period (Parry, 2013). While they do possess a mobile “crawler” form during early stages of development, and small‐bodied, wingless arthropods can achieve passive dispersal across significant distances (Washburn & Frankie, 1981, Meineke & Davies, 2019), their mobility likely remains somewhat constrained compared to other *Citrus* pests. This stage‐dependent variation in dispersal ability creates only brief windows for movement from surrounding landscapes into *Citrus* cropland, likely contributing to the neutral effects of natural and urban land use on scale insect populations.

We also expected that (ii) the one predator species would demonstrate responses that were the inverse of those predicted for pests, with natural land increasing predator density and urban land decreasing it (Korányi et al., 2022; Rusch et al., 2016). We found partial support for these predictions in that higher proportions of crop‐adjacent urban land were significantly associated with lower *Euseius* mite densities. However, contrary to our expectations, *Euseius* mite densities declined with increasing surrounding natural area. The negative association of natural land with *Euseius* mite densities is particularly surprising, because in San Joaquin Valley *Citrus*, the genus consists primarily of the native species *Euseius tularensis*, and native predators typically find suitable alternative host plants and associated prey in surrounding natural habitats (Leimu et al., 2012; Pearse & Rosenheim, 2020). While the specific mechanisms underlying this negative relationship are unknown, it is possible that surrounding natural habitats provide more appealing alternate prey or host plants than *Citrus* fields, diverting these predators away from cropland (Möth et al., 2021; Tixier, 2018; Tscharntke et al., 2016). Future work should investigate whether *Euseius* movement patterns between natural areas and crop fields support this resource‐driven explanation, and when this pattern might extend to other predator taxa in perennial agroecosystems.

### Effects of surrounding landscapes on economic measures of *Citrus* production

For growers to adopt research‐driven recommendations, it is critical to go beyond the assessment of landscape effects on pest pressure and examine landscape effects on downstream economic measures of crop production (Chaplin‐Kramer et al., 2019; Guerry et al., 2015). We therefore assessed how surrounding natural and urban land affected three metrics of direct economic importance for growers: total pesticide use, fruit quality, and total fruit yield. We found significant effects of surrounding landscapes on two relevant economic metrics: total pesticide use and total fruit yield. More natural land was significantly associated with an increase in total pesticide use and a reduction in total fruit yield, while urban land was associated with a decrease in total pesticide use.

Interestingly, the effects of natural and urban land on total pesticide use did not reflect density results for our focal arthropod species, where both land use types were associated with increased densities of only one pest (citrus thrips). This discrepancy likely stems from our study's focus on the most prevalent pest species, whereas the measure of total pesticide use encompasses a broader scope of pests found in *Citrus*, including ants, earwigs, snails, cutworms, and the orange dog caterpillar. Consequently, our total pesticide use analysis provides a more comprehensive picture of how surrounding landscape composition influences overall pest dynamics in *Citrus* agroecosystems. The finding that more surrounding urban land is associated with decreased pesticide use, despite mixed effects on pest densities, might be explained by regulatory practices. As the harmful effects of pesticides on human health are well documented (Larsen et al., 2017), pesticide applications near urban and populated areas, especially near schools, are highly regulated (California Department of Pesticide Regulation, 2017). These restrictions likely drive the reduced pesticide use trend demonstrated in *Citrus* fields adjacent to urban landscapes, independent of observed pest pressure.

Another adverse outcome for farmers was the association of natural land with a strong reduction (up to 55%) in total fruit yield. Some studies have demonstrated, however, that other measures of crop yield, such as yield stability, can increase in response to surrounding natural land, even as relative yield decreases (Redhead et al., 2020). We suggest that the decrease in yield of citrus groves with more nearby natural land is unlikely to be a reflection of pest pressure, because growers rarely allow pest populations to build up beyond the point of yield loss. Instead, crop yield has been tightly linked to soil quality (D'Hose et al., 2014; Suresh & Nagesh, 2015), a feature of the landscape that is likely to vary non‐randomly across the agricultural matrix, because areas with high soil quality are more likely to be converted to farmland (Poveda et al., 2018; Verburg et al., 2004). As a result, areas at the margins of cropland (agricultural–natural interfaces) may have lower soil quality on average, resulting in lower crop yields. Overall, our results neither support nor oppose efforts to retain natural habitat remnants or minimize urban development near cropland for the purpose of enhancing *Citrus* production for growers. Instead, our results suggest that growers can design their pest management programs by focusing on within‐field conditions, presumably a simpler task in most cases.

## CONCLUSION

Within each agroecosystem, there exists only one physical landscape for managing the entire community of arthropods. Effective management at the landscape scale would aim to influence all community members (or at least the most damaging species) in a way that provides net benefits to growers. In most cases, the lack of sufficient data for the many pest species active within each agroecosystem will challenge any efforts to optimize these landscape‐level management decisions. Here, where the data are available and uniquely suited to examine these questions, our results highlight how unlikely it is for surrounding land use to affect an entire suite of crop pests and direct economic outcomes for growers in a consistent manner.

The difficult task of designing a landscape to improve pest management becomes even more challenging when we begin to account for how the landscape varies across time and space, often in unpredictable ways. For example, an agro‐landscape can change dramatically within and across years, with annual crops being planted, harvested, or rotated to other crop types, facilitating or inhibiting temporal continuity for some pest and natural enemy species (Iuliano & Gratton, 2020). Across years, longer‐term processes such as warming temperatures can differentially affect crops and their associated pests and natural enemies such that the benefits of a particular landscape design could change (Welch & Harwood, 2014). Ultimately, a robust agricultural landscape design for conservation biocontrol would be difficult to define, as it would need to produce a net beneficial outcome for growers across all crop pests, beneficials, and crop plants across the landscape and through time. Instead, we conclude that in most cases, growers should design their pest management programs by focusing on within‐field conditions, employing strategies to enhance biological control and suppress pest buildup at the local scale. Common practices include strategic cover cropping (Beaumelle et al., 2021; Rowen et al., 2022), establishment of native flowering plants along field margins (Balzan & Moonen, 2014; Obanyi et al., 2024), and maintaining appropriate crop sanitation practices such as eradicative tree pruning (Matias et al., 2023; Svihra, 1994).

Taken together, the results of our study do not demonstrate clear support for the retention of natural habitat or the minimization of urban land near cropland for the purpose of decreasing pest densities, enhancing conservation biocontrol or benefitting downstream crop production. However, agriculture continues to intensify to meet the demands of a growing global population, and responsible stewardship of developed land is increasingly critical for the retention of biodiversity (Dudley & Alexander, 2017). To reconcile social, environmental, and economic objectives, we must identify, preserve, and create new natural landscape elements that bolster ecosystem services across productive agricultural landscapes (Bommarco et al., 2013; Landis, 2017; Zymaroieva et al., 2021). In a time of expanding urbanization and rapid habitat loss for many species, we urge the design of working landscapes to strike a balance between the needs of agricultural production and the conservation of biodiversity even when its effects are not immediately demonstrated.

## CONFLICT OF INTEREST STATEMENT

The authors declare no conflicts of interest.

## Supporting information


Appendix S1.

Appendix S2.

Appendix S3.

Appendix S4.

Appendix S5.

## Data Availability

Data (Lippey et al., 2025) is available in Dryad at https://doi.org/10.5061/dryad.08kprr5cb.
