# Supplementary material for: One landscape does not fit all: Diverse arthropod responses to land use
Source: Ecol Appl. 2025 Nov 12;35(7):e70132. doi: 10.1002/eap.70132 (PMC12611504; doi:10.1002/eap.70132)
Supplement: Supplementary file 1 — Appendix S1. [file EAP-35-e70132-s002.pdf]

**Supporting Information**

**One landscape does not fit all: Diverse arthropod responses to land use**

**Mia K. Lippey, Jay A. Rosenheim, Daniel Paredes, Daniel S. Karp, Sara E. Emery, Rebecca Chaplin-Kramer, Richard Sharp, Emily K. Meineke**

*Ecological Applications*

**Appendix S1**

Table S1: Common native and non-native plant families and genera documented in the San Joaquin Valley.

| Land use association | Plants present (family or genus) |
|---|---|
| Natural habitats | *Achillea, Aquilegia, Aristolochia, Aster, Berberis, Bouteloua, Calycanthus, Carpinteria, Ceanothus, Cercis, Cercocarpus, Epilobium, Eriogonum, Festuca, Heteromeles, Heuchera, Iris, Isomeris, Juglans, Lessingia, Muhlenbergia, Penstemon, Quercus, Rhamnus, Ribes, Rosa, Rubus, Salix, Salvia, Simmondsia, Solidago, Umbellularia, Viguiera, Viola, Vitis, Woodwardia*, and more |
| Urban streets and gardens | Arecaceae, *Arundo*, *Brassica*, *Calendula*, *Camellia*, *Capsicum*, *Centaurea*, *Chrysanthemum*, *Citrullus*, *Citrus*, *Cucurbita*, *Dianthus*, *Digitalis*, *Dimorphotheca*, *Eucalyptus*, *Iberis*, *Lavandula*, *Malus*, *Myrtus*, *Nerium*, *Prunus*, *Punica*, *Pyrus*, *Ricinus*, *Scabiosa*, *Solanum*, *Tamarix*, *Ulmus*, *Vinca*, and more |

Table S2: Host ranges of focal arthropod species included in this study. All species are generalists with several food sources. Six species are herbivorous (citricola scale, California red scale, citrus thrips, citrus red mite, cottony cushion scale, and citrus peelminer), one species is omnivorous (fork-tailed bush katydid), and the beneficial Euseius mite is predatory.

| Arthropod species | Feeding guild | Number of documented host plant species | Plant types (family or genus) |
|---|---|---|---|

| | | | |
|---|---|---|---|
| Fork-tailed bush katydid (*Scudderia furcata*) | Generalist omnivore | No information published | *Chrysanthemum, Citrus, Dimorphotheca, Morus, Prunus, Rosa, Viola*, and more |
| The citricola scale (*Coccus pseudomagnoliarum*) | Generalist herbivore | 20 | *Nerium, Berberis, Celtis, Juglans, Laurus, Clerodendrum, Punica, Myrtus, Rhamnus, Citrus, Ulmus, Zelkova*, and more |
| The California red scale (*Aonidiella aurantii*) | Generalist herbivore | 77 | *Abelmoschus, Acacia, Aleurites, Annona, Araucaria, Ardisia,* Arecaceae, *Artocarpus, Asparagus, Bauhinia, Broussonetia, Buxus, Camellia, Capsicum, Carica, Casuarina, Ceiba, Chaenomeles, Cinnamomum, Citrus, Cocos, Cucurbita, Cycas, Erythrina, Eucalyptus, Ficus, Gardenia, Gossypium, Grevillea, Hedera, Hibiscus, Ilex, Jasminum, Jodina, Juglans, Laurus, Ligustrum, Malus, Mangifera, Melia, Morus, Musa, Nerium, Passiflora, Phoenix, Pistacia, Psidium, Punica, Ricinus, Rosa, Rubus, Salix, Schefflera, Sida, Tamarindus, Vitis, Yucca, Ziziphus,* and more |
| The citrus thrips (*Scirtothrips citri*) | Generalist herbivore | 35 | *Carya, Citroncirus, Citrus, Coffea, Dahlia, Larrea, Magnolia, Myrtus, Quercus, Rhizophora, Rhus, Rosa, Salix, Schinus, Simmondsia, Umbellularia, Vaccinium, Vitis,* and more |
| The citrus red mite (*Panonychus citri*) | Generalist herbivore | 111 | Arecaceae, *Prunus, Pyrus, Ricinus, Vitis,* and more |
| The cottony cushion scale (*Icerya purchasi*) | Generalist herbivore | 25 | *Acacia, Acalypha, Arachis, Calliandra, Citrus, Cosmos, Cussonia, Eucalyptus, Hibiscus, Laurus, Liquidambar, Magnolia, Mangifera, Mimosa, Morus, Nandina, Parrotia, Pelargonium, Pittosporum, Psidium, Salvia,* and more |

| | | | |
|---|---|---|---|
| The citrus peelminer (*Marmara gulosa*) | Generalist herbivore | 69 | *Capsicum, Citrus, Cucurbita, Gossypium, Malus, Prunus,* and more |
| *Euseius* mite | Generalist predator | No information published | *Citrus,* any other plant species that host prey |
