# Supplementary material for: One landscape does not fit all: Diverse arthropod responses to land use
Source: Ecol Appl. 2025 Nov 12;35(7):e70132. doi: 10.1002/eap.70132 (PMC12611504; doi:10.1002/eap.70132)
Supplement: Supplementary file 2 — Appendix S2. [file EAP-35-e70132-s004.pdf]

Supporting Information

One landscape does not fit all: Diverse arthropod responses to land use

Mia K. Lippey, Jay A. Rosenheim, Daniel Paredes, Daniel S. Karp, Sara E. Emery, Rebecca Chaplin-Kramer, Richard Sharp, Emily K. Meineke

Ecological Applications

Appendix S2

Detailed descriptions of sampling methods for focal arthropod species in *Citrusformatics.*

1) The fork-tailed bush katydid (*Scudderia furcata* (Brunner von Wattenwyl) [Orthoptera: Tettigoniidae], *N* = 792) is a native, key pest of California citrus. It is omnivorous, feeding on the young leaves and fruits of host plants and also on small arthropods. Chewing damage by nymphal katydids on young citrus fruits develops into deep scars on the fruit rind at the time of harvest (UC IPM Pest Management Guidelines, n.d.). Pest control advisors monitor katydids in California citrus for 60 days post-petalfall and determine presence/absence by observing a small, defined area of foliage. For all arthropod analyses, we used a weighted average of observations.

2) The citricola scale (*Coccus pseudomagnoliarum* (Kuwana) [Hemiptera: Coccidae], *N* = 961) is a non-native pest. It is a generalist herbivore that has been documented on at least 20 different species of host plants (Stathas & Karipidis, 2020). Citriciola scale causes damage by feeding on vegetative plant structures and by excreting honeydew, which supports the growth of sooty mold. Citricola scale reduces tree vigor and fruit set and can cause downgrading of fruit quality at the time of harvest (UC IPM Pest Management Guidelines, n.d.). Pest control advisors monitor citricola scale in California citrus using presence/absence sampling on single leaves.

3) The California red scale (*Aonidiella aurantii* (Maskell) [Hemiptera: Diaspididae], *N* =793) is a non-native armored scale. It is a hyper-generalist herbivore that has been

documented on at least 77 different species of host plants (Borchsenius, 1966). California red scale causes damage by feeding on all above-ground parts of the citrus tree. Severe infestations cause leaf yellowing and dieback of branches (UC IPM Pest Management Guidelines, n.d.). We analyzed data on California red scale presence/absence on fruit at harvest, quantified per bin sample (ca. 100 fruit checked per bin).

4) The citrus thrips (*Scirtothrips citri* (Moulton) [Thysanoptera: Thripidae], N = 2205 field-years) is a native pest. It is a generalist herbivore that has been documented on at least 35 different species of host plants (EPPO Global Database). Larval stages cause damage by piercing epidermal cells of the citrus rind, resulting in superficial silvery rind scarring at the time of harvest (UC IPM Pest Management Guidelines, n.d.). Pest control advisors use presence/absence sampling of 25-150 small developing fruitlets to monitor citrus thrips in California citrus throughout the first two months post-petalfall.

5) The citrus red mite (*Panonychus citri* (McGregor) [Acari: Tetranychidae], *N* = 1350) is a non-native spider mite pest. It is a hyper-generalist herbivore that has been documented on at least 111 different species of host plants (Zhang 2003; Vacante 2010; Hoy 2011). Citrus red mite causes damage by feeding on leaves, creating stippling. Severe infestations result in the death of leaves and twigs, and occasionally stippling of the fruit itself (UC IPM Pest Management Guidelines, n.d.). Pest control advisors monitor citrus red mites in California citrus using presence/absence sampling on single tree leaves across a four month period between March and June.

6) The cottony cushion scale (*Icerya purchasi* (Maskell) [Hemiptera: Monophlebidae], *N* = 750) is a non-native pest. It is a generalist herbivore that has been documented on at least 25 different species of host plants (EPPO Global Database). Cottony cushion scale causes damage feeding on all above-ground parts of the tree. A severe infestation eventually leads to reduction in tree vigor (UC IPM Pest Management Guidelines, n.d.). Pest control advisors monitor cottony cushion scale in California citrus using presence/absence sampling on 60-cm portions of tree trunks and inner branches.

7) The citrus peelminer (*Marmara gulosa* (Guillèn and Davis) [Lepidoptera: Gracillariidae], N = 774) is a non-native pest. It is a generalist herbivore that has been documented on at least 69 different species of host plants (UC ANR, 2008). Larval stages cause damage by tunneling beneath the surface of the fruit rind, resulting in downgraded fruit at the time of harvest (UC IPM Pest Management Guidelines, n.d.). Pest control advisors in California citrus rarely sample for citrus peelminer during the growing season, but the peelminer leaves a distinct damage pattern easily identifiable at harvest. For our pest density analysis, we used the number of fruit damaged at harvest, quantified per bin sample (ca. 100 fruit checked per bin).|

8) Predatory mites in the genus *Euseius* ((Congdon) [Acarina: Phytoseiidae], *N* = 335), which predominantly comprise the native species *Euseius tularensis* in the Central Valley (Grafton-Cardwell *et al.,* 2020), provide biological control for many pest species in citrus. *Euseius* mites feed on nymphs of scale insects and other soft-bodied arthropods including whiteflies, aphids, mites, and thrips (UC IPM Pest Management Guidelines, n.d.). Pest control advisors monitor *Euseius* mites in California citrus by counting the number of mites on five-leaf branch terminals. Five terminals were sampled per 20-acre block, and the average number of mites per terminal was taken and rounded to the nearest integer value.
