# Supplementary material for: One landscape does not fit all: Diverse arthropod responses to land use
Source: Ecol Appl. 2025 Nov 12;35(7):e70132. doi: 10.1002/eap.70132 (PMC12611504; doi:10.1002/eap.70132)
Supplement: Supplementary file 3 — Appendix S3. [file EAP-35-e70132-s003.pdf]

Supporting Information

One landscape does not fit all: Diverse arthropod responses to land use

Mia K. Lippey, Jay A. Rosenheim, Daniel Paredes, Daniel S. Karp, Sara E. Emery, Rebecca Chaplin-Kramer, Richard Sharp, Emily K. Meineke

Ecological Applications

Appendix S3

Table S1 (next page): Summary statistics of all land use types surrounding focal *Citrus* fields across the four spatial scales examined (500m, 1km, 2km, and 4km buffer radii), expressed as proportions of area within circular buffers. We dropped two land use categories, other cropland and other non-cropland, from all models to avoid issues of non-independence between land use variables. To create the urban land use category, we summed four land use types identified by the USGS NLCD: developed open space; developed low intensity; developed medium intensity; and developed high intensity. To create the natural land use category, we summed four land use types identified by the USGS NLCD: shrub/scrub; herbaceous; emergent herbaceous wetlands; and woody wetlands. To create the citrus land category, we summed two land use types identified by the USDA CDL: oranges and *Citrus*. To create the other cropland category, we subtracted the USDA CDL *Citrus* area from the USGA NLCD category of cultivated crops. Finally, to create the other noncropland category, we summed four land use types identified by the USGS NLCD: open water; barren land; and hay/pasture.

Land use type	Spatial scale	Mean	Standard error
Natural	500m	0.11	0.00
	1km	0.16	0.01
	2km	0.20	0.01
	4km	0.26	0.01
Urban	500m	0.04	0.00
	1km	0.06	0.00
	2km	0.08	0.00
	4km	0.09	0.00
Citrus	500m	0.67	0.01
	1km	0.43	0.01
	2km	0.11	0.00
	4km	0.02	0.00
Other cropland	500m	0.16	0.01
	1km	0.32	0.01
	2km	0.57	0.01
	4km	0.59	0.01
Other non-cropland	500m	0.02	0.00
	1km	0.03	0.00
	2km	0.03	0.00
	4km	0.03	0.00

Table S2: Summary statistics of all other model variables. Response variables include pest density, natural enemy density, targeted pesticide applications, total pesticide use, fruit quality, and total fruit yield. We also included summary statistics for the covariate field size.

Model variable	Mean	Standard error
Fork-tailed bush katydid density (% infestation)	0.02	0.00
Citricola scale density (% infestation)	0.03	0.00
California red scale density (abundance)	1.68	0.19
Citrus thrips density (% infestation)	0.03	0.00
Citrus red mite density (% infestation)	0.16	0.01
Cottony cushion scale density (% infestation)	0.03	0.00
Citrus peelminer density (abundance)	0.13	0.03
*Euseius*mite density (abundance)	3.47	0.08
Pesticide applications targeting fork-tailed bush katydid (# of sprays)	0.38	0.01
Pesticide applications targeting citricola scale (# of sprays)	0.10	0.01
Pesticide applications targeting California red scale (# of sprays)	0.81	0.02
Pesticide applications targeting citrus thrips (# of sprays)	0.95	0.02
Pesticide applications targeting citrus red mite (# of sprays)	0.64	0.02
Total pesticide use (total # of pesticide sprays)	9.83	0.14
Fruit quality (continuous rank from 0-2)	1.32	0.01
Total fruit yield (kg/ha)	27916.25	433.52
Field size (ha)	11.04	0.16
