# Supplementary material for: One landscape does not fit all: Diverse arthropod responses to land use
Source: Ecol Appl. 2025 Nov 12;35(7):e70132. doi: 10.1002/eap.70132 (PMC12611504; doi:10.1002/eap.70132)

Supporting Information

One landscape does not fit all: Diverse arthropod responses to land use

Mia K. Lippey, Jay A. Rosenheim, Daniel Paredes, Daniel S. Karp, Sara E. Emery, Rebecca Chaplin-Kramer, Richard Sharp, Emily K. Meineke

Ecological Applications

Appendix S4

Table S1: Moran's I analysis results per species, comparing models with and without the subregion random effect. Across the eight insect species models, all showed significant spatial autocorrelation in their residuals when the subregion random effect was omitted. After including the subregion random effect, only five species still showed significant spatial autocorrelation, with a mean reduction in Moran's I values of 0.072. This analysis confirms that inclusion of the spatial random effect substantially mitigates spatial dependencies in the data. For the five species that still showed significant spatial autocorrelation after including the spatial random effect, the magnitude of autocorrelation was substantially reduced. This suggests that while our approach does not completely eliminate spatial dependencies for all species, it significantly improves the model by accounting for a substantial portion of the spatial structure in the data.

Species model	Moran's I (no subregion random effect term included)	p-value (no subregion random effect term included)	Moran's I (subregion random effect term included)	p-value (subregion random effect term included)
Fork-tailed bush katydid	0.086	0.000	-0.004	0.570
Citricola scale	0.338	0.000	0.135	0.000
California red scale	0.031	0.022	0.017	0.129
Citrus thrips	0.095	0.000	0.055	0.000
Citrus red mite	0.040	0.002	0.016	0.118
Cottony cushion scale	0.194	0.000	0.112	0.000
Citrus peelminer	0.042	0.000	0.017	0.037
Euseius mite	0.208	0.000	0.114	0.000

Figure S1: Visual representation of Moran's I analysis results. The dotted line represents where the Moran's I values would be equal with and without the spatial random effect (i.e., no improvement). Species points below this line indicate that the Moran's I value is lower with the spatial random effect than without it. We show that for every insect species, including the spatial random effect (subregion) reduced the spatial autocorrelation in the model residuals.

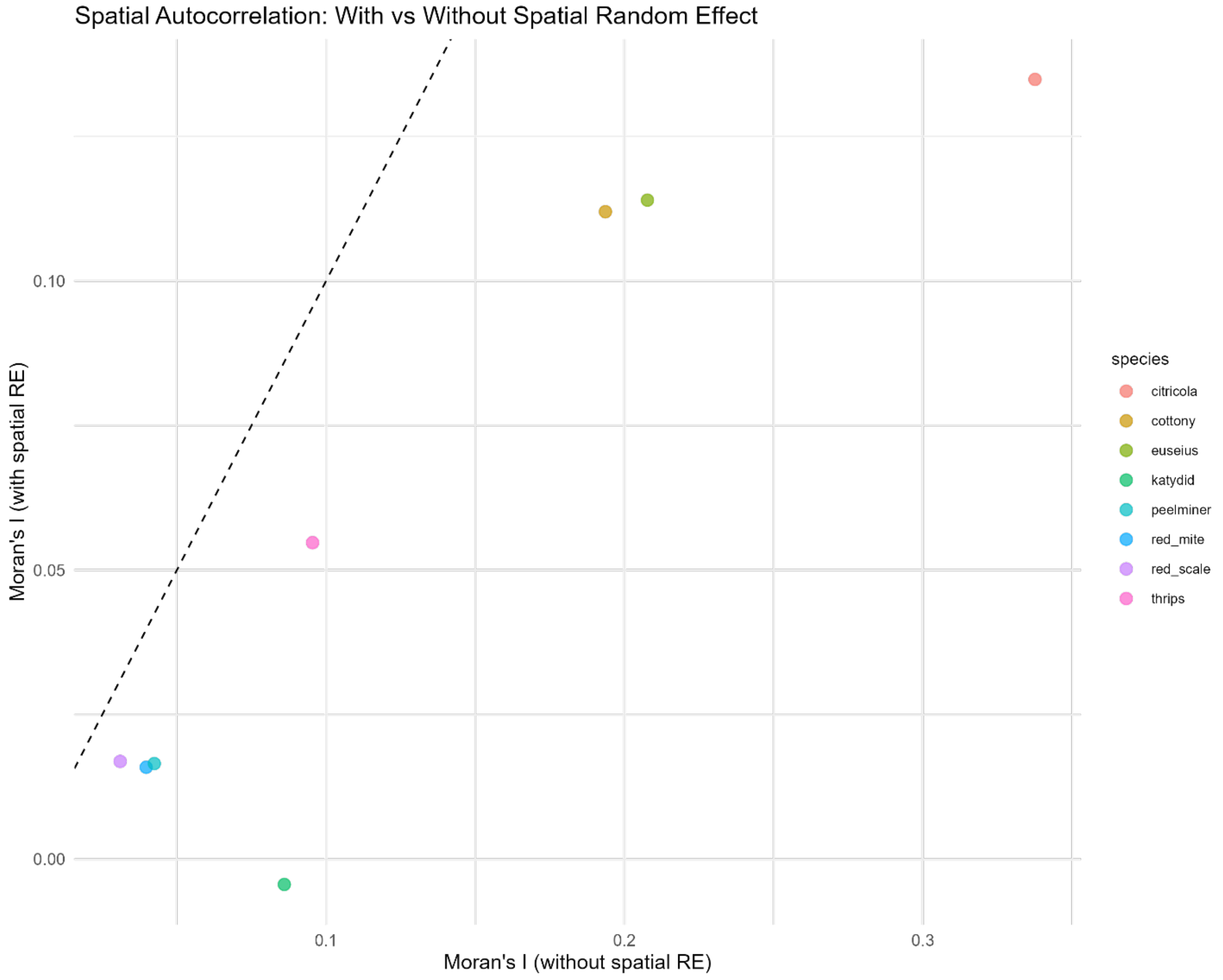

Supplement: Supplementary file 4 — Appendix S4. [file EAP-35-e70132-s005.pdf]
