# Supplementary material for: One landscape does not fit all: Diverse arthropod responses to land use
Source: Ecol Appl. 2025 Nov 12;35(7):e70132. doi: 10.1002/eap.70132 (PMC12611504; doi:10.1002/eap.70132)
Supplement: Supplementary file 5 — Appendix S5. [file EAP-35-e70132-s001.pdf]

Supporting Information

One landscape does not fit all: Diverse arthropod responses to land use

Mia K. Lippey, Jay A. Rosenheim, Daniel Paredes, Daniel S. Karp, Sara E. Emery, Rebecca Chaplin-Kramer, Richard Sharp, Emily K. Meineke

Ecological Applications

Appendix S5

Table S1 (next page): Modeling results for the effect of natural and urban land on densities of eight focal arthropod species across four spatial scales (buffer radii of 500m, 1km, 2km, and 4km). Bolded text indicates the result for which the spatial scale minimized the AIC value of the model for each species. For the zero-inflated beta models (fork-tailed bush katydid, citricola scale, citrus red mite, cottony cushion scale, and citrus thrips), we are reporting the coefficients from the beta component (not the zero-inflation component), which estimate the effect of predictors on the mean of the distribution for non-zero values. These coefficients represent the change in the logit-transformed mean of the beta distribution per unit change in the predictor. For the zero-inflated Tweedie (California red scale and citrus peelminer) and simple Tweedie models (the predatory *Euseius* mite), the coefficients represent the effect of predictors on the log-transformed mean of the Tweedie distribution.

Species	Scale	Land use type	β estimate	Standard error	Z-value	p-value	N	AIC
Fork-tailed bush katydid (*Scudderia furcata*)	500m	urban	0.027	0.054	0.498	0.618	714	-1281.030
		natural	0.024	0.089	0.271	0.787	714	-1281.030
	1km	urban	0.012	0.052	0.234	0.815	714	-1279.377
		natural	0.052	0.105	0.497	0.619	714	-1279.377
	**2km**	**urban**	**0.097**	**0.054**	**1.788**	**0.074**	**714**	**-1283.946**
		**natural**	**0.006**	**0.078**	**0.080**	**0.936**	**714**	**-1283.946**
	4km	urban	0.102	0.069	1.473	0.141	714	-1281.437
		natural	0.028	0.087	0.327	0.744	714	-1281.437
Citricola scale (*Coccus pseudomagnoliarum*)	500m	urban	0.002	0.064	0.031	0.975	766	471.306
		natural	0.020	0.142	0.138	0.891	766	471.306
	1km	urban	-0.020	0.071	-0.281	0.779	766	470.818
		natural	0.039	0.133	0.290	0.772	766	470.818
	2km	urban	-0.084	0.078	-1.077	0.281	766	471.746
		natural	-0.065	0.126	-0.517	0.605	766	471.746
	**4km**	**urban**	**-0.185**	**0.114**	**-1.616**	**0.106**	**766**	**470.063**
		**natural**	**-0.146**	**0.139**	**-1.054**	**0.292**	**766**	**470.063**
California red scale (*Aonidiella aurantii*)	500m	urban	-0.070	0.110	-0.634	0.526	765	3180.437
		natural	0.649	0.204	3.178	0.001	765	3180.437
	1km	urban	-0.181	0.110	-1.636	0.102	765	3185.339
		natural	-0.156	0.231	-0.675	0.500	765	3185.339
	2km	urban	-0.169	0.147	-1.150	0.250	765	3180.316
		natural	-0.418	0.321	-1.304	0.192	765	3180.316
	**4km**	**urban**	**-0.431**	**0.190**	**-2.268**	**0.023**	**765**	**3178.869**
		**natural**	**-0.229**	**0.292**	**-0.784**	**0.433**	**765**	**3178.869**
Citrus thrips (*Scirtothrips citri*)	500m	urban	0.022	0.027	0.829	0.407	1424	-5876.679
		natural	0.112	0.052	2.165	0.030	1424	-5876.679
	1km	urban	0.010	0.027	0.368	0.713	1424	-5883.818
		natural	0.243	0.059	4.093	0.000	1424	-5883.818
	2km	urban	0.034	0.034	0.998	0.318	1424	-5889.560
		natural	0.320	0.079	4.048	0.000	1424	-5889.560
	**4km**	**urban**	**0.121**	**0.047**	**2.585**	**0.010**	**1424**	**-5893.047**
		**natural**	**0.302**	**0.070**	**4.292**	**0.000**	**1424**	**-5893.047**
Citrus red mite (*Panonychus citri*)	500m	urban	0.004	0.040	0.097	0.923	1130	-32.115
		natural	-0.126	0.076	-1.657	0.098	1130	-32.115
	1km	urban	0.035	0.040	0.857	0.392	1130	-33.221
		natural	-0.154	0.079	-1.960	0.050	1130	-33.221
	**2km**	**urban**	**0.023**	**0.049**	**0.469**	**0.639**	**1130**	**-40.928**
		**natural**	**-0.271**	**0.091**	**-2.968**	**0.003**	**1130**	**-40.928**
	4km	urban	-0.116	0.075	-1.535	0.125	1130	-37.042

		natural	-0.234	0.091	-2.576	0.010	1130	-37.042
Cottony cushion scale (*Icerya purchasi*)	**500m**	**urban**	**-0.031**	**0.098**	**-0.319**	**0.750**	**592**	**363.332**
		**natural**	**-0.198**	**0.205**	**-0.963**	**0.335**	**592**	**363.332**
	1km	urban	-0.035	0.098	-0.353	0.724	592	366.411
		natural	0.087	0.235	0.369	0.712	592	366.411
	2km	urban	-0.036	0.106	-0.342	0.733	592	364.407
		natural	-0.179	0.185	-0.966	0.334	592	364.407
	4km	urban	0.063	0.139	0.452	0.651	592	363.424
		natural	-0.209	0.192	-1.086	0.278	592	363.424
Citrus peelminer (*Marmara gulosa*)	500m	urban	-0.001	0.093	-0.010	0.992	752	-608.012
		natural	-0.044	0.195	-0.225	0.822	752	-608.012
	1km	urban	-0.190	0.090	-2.111	0.035	752	-619.192
		natural	-0.677	0.094	-7.182	0.000	752	-619.192
	2km	urban	-0.278	0.112	-2.489	0.013	752	-636.815
		natural	-0.773	0.154	-5.015	0.000	752	-636.815
	**4km**	**urban**	**-0.416**	**0.132**	**-3.164**	**0.002**	**752**	**-639.360**
		**natural**	**-0.902**	**0.150**	**-6.028**	**0.000**	**752**	**-639.360**
Euseius mite (*Euseius spp.*)	500m	urban	-0.005	0.042	-0.124	0.901	346	1209.522
		natural	0.019	0.030	0.650	0.516	346	1209.522
	1km	urban	-0.062	0.039	-1.566	0.117	346	1206.756
		natural	0.010	0.031	0.309	0.757	346	1206.756
	2km	urban	-0.022	0.036	-0.627	0.531	346	1204.284
		natural	-0.074	0.033	-2.212	0.027	346	1204.284
	**4km**	**urban**	**-0.127**	**0.043**	**-2.926**	**0.003**	**346**	**1191.592**
		**natural**	**-0.140**	**0.038**	**-3.717**	**0.000**	**346**	**1191.592**

Table S2 (next page): Modeling results for the effect of natural and urban land on targeted pesticide applications for eight focal arthropod species across four spatial scales (buffer radii of 500m, 1km, 2km, and 4km). Bolded text indicates the result that matches the spatial scale of the density model that minimized the AIC value for each species. For the Poisson models (all targeted pesticide models), the Poisson family in glmmTMB automatically implements a log link function. The slopes in these models represent the effect on the log-transformed mean count of pesticide applications per unit change in the predictor. These relationships are linear on the log scale.

Species	Scale	Land use type	β estimate	Standard error	Z-value	p-value	N	AIC
Fork-tailed bush katydid (*Scudderia furcata*)	500m	urban	-0.031	0.046	-0.675	0.500	1526	2030.472
		natural	0.155	0.049	3.196	0.001	1526	2030.472
	1km	urban	-0.023	0.047	-0.498	0.619	1526	2029.366
		natural	0.166	0.050	3.347	0.001	1526	2029.366
	**2km**	**urban**	**-0.039**	**0.049**	**-0.802**	**0.423**	**1526**	**2032.050**
		**natural**	**0.114**	**0.048**	**2.349**	**0.019**	**1526**	**2032.050**
	4km	urban	-0.020	0.055	-0.365	0.715	1526	2030.244
		natural	0.142	0.057	2.499	0.012	1526	2030.244
Citricola scale (*Coccus pseudomagnoliarum*)	500m	urban	0.064	0.084	0.764	0.445	1526	639.426
		natural	-0.130	0.158	-0.823	0.410	1526	639.426
	1km	urban	0.167	0.071	2.355	0.019	1526	633.755
		natural	-0.104	0.109	-0.951	0.342	1526	633.755
	2km	urban	0.202	0.074	2.730	0.006	1526	633.986
		natural	-0.036	0.105	-0.344	0.731	1526	633.986
	**4km**	**urban**	**0.200**	**0.078**	**2.571**	**0.010**	**1526**	**633.300**
		**natural**	**-0.030**	**0.109**	**-0.279**	**0.780**	**1526**	**633.300**
California red scale (*Aonidiella aurantii*)	500m	urban	0.037	0.031	1.199	0.231	1526	3084.925
		natural	-0.008	0.067	-0.120	0.905	1526	3084.925
	1km	urban	0.046	0.034	1.354	0.176	1526	3084.728
		natural	-0.077	0.083	-0.924	0.356	1526	3084.728
	2km	urban	-0.001	0.045	-0.033	0.974	1526	3092.679
		natural	-0.171	0.124	-1.375	0.169	1526	3092.679
	**4km**	**urban**	**-0.202**	**0.062**	**-3.291**	**0.001**	**1526**	**3076.618**
		**natural**	**-0.078**	**0.075**	**-1.049**	**0.294**	**1526**	**3076.618**
Citrus thrips (*Scirtothrips citri*)	500m	urban	0.017	0.026	0.667	0.505	1526	3163.124
		natural	0.034	0.031	1.083	0.279	1526	3163.124
	1km	urban	0.043	0.027	1.612	0.107	1526	3159.926
		natural	0.056	0.031	1.798	0.072	1526	3159.926
	2km	urban	0.043	0.028	1.505	0.132	1526	3160.643
		natural	0.054	0.031	1.779	0.075	1526	3160.643
	**4km**	**urban**	**0.064**	**0.031**	**2.047**	**0.041**	**1526**	**3159.392**
		**natural**	**0.068**	**0.035**	**1.951**	**0.051**	**1526**	**3159.392**
Citrus red mite (*Panonychus citri*)	500m	urban	-0.040	0.036	-1.098	0.272	1526	2636.304
		natural	-0.066	0.091	-0.727	0.467	1526	2636.304
	1km	urban	-0.018	0.037	-0.480	0.631	1526	2634.962
		natural	-0.139	0.088	-1.578	0.115	1526	2634.962
	**2km**	**urban**	**-0.053**	**0.047**	**-1.126**	**0.260**	**1526**	**2618.500**
		**natural**	**-0.110**	**0.099**	**-1.111**	**0.267**	**1526**	**2618.500**
	4km	urban	-0.147	0.070	-2.108	0.035	1526	2626.059
		natural	-0.114	0.110	-1.043	0.297	1526	2626.059

Table S3: Modeling results for the effect of natural and urban land on downstream economic measures of crop production (total pesticide use, fruit quality, and total fruit yield) across four spatial scales (buffer radii of 500m, 1km, 2km, and 4km). Bolded text indicates the result for which the spatial scale minimized the AIC value of the model for each response variable. For the Poisson models (total pesticide use), the beta models (fruit quality), and the simple Tweedie models (total fruit yield), the coefficients represent the effects of predictors as described above for pest/predator density models and targeted pesticide models.

Economic measure of *Citrus* production	Spatial scale	Land use type	β estimate	Standard error	Z-value	p-value	N	AIC
Total pesticide use	500m	urban	0.615	0.447	1.375	0.169	1937	10889.230
		natural	0.248	0.150	1.648	0.099	1937	10889.230
	**1km**	**urban**	**0.655**	**0.393**	**1.667**	**0.095**	**1937**	**10887.529**
		**natural**	**-0.014**	**0.157**	**-0.087**	**0.931**	**1937**	**10887.529**
	2km	urban	-2.279	0.620	-3.673	0.000	1937	10865.450
		natural	0.861	0.223	3.853	0.000	1937	10865.450
	4km	urban	-2.525	0.640	-3.946	0.000	1937	10876.189
		natural	-0.006	0.219	-0.029	0.977	1937	10876.189
Fruit quality	500m	urban	-0.226	0.994	-0.227	0.820	1483	-1137.653
		natural	0.217	0.286	0.761	0.447	1483	-1137.653
	**1km**	**urban**	**0.817**	**0.904**	**0.904**	**0.366**	**1483**	**-1147.414**
		**natural**	**-0.144**	**0.303**	**-0.475**	**0.635**	**1483**	**-1147.414**
	2km	urban	-1.420	1.184	-1.200	0.230	1483	-1136.326
		natural	-0.722	0.425	-1.700	0.089	1483	-1136.326
	4km	urban	0.306	1.230	0.249	0.804	1483	-1137.921
		natural	-0.363	0.343	-1.058	0.290	1483	-1137.921
Total fruit yield	500m	urban	-0.023	0.029	-0.776	0.438	1646	35931.765
		natural	-0.027	0.050	-0.536	0.592	1646	35931.765
	1km	urban	-0.003	0.028	-0.088	0.930	1646	35917.227
		natural	-0.228	0.054	-4.238	0.000	1646	35917.227
	**2km**	**urban**	**-0.032**	**0.036**	**-0.880**	**0.379**	**1646**	**35922.219**
		**natural**	**-0.357**	**0.078**	**-4.599**	**0.000**	**1646**	**35922.219**
	4km	urban	-0.051	0.048	-1.077	0.281	1646	35918.380
		natural	-0.324	0.081	-3.979	0.000	1646	35918.380
